# Researches on Emphysema of the Lungs

**Published:** 1838-03-28

**Authors:** 


**Researches on Emphysema of the Lungs.** *By* M. Louis, Physician of La Pitie, &c. &c. &c. *Translated by* T. Stewardson, Jr., M. D., Physician to the Blockley Hospital, &c. &c. &c. *Philadelphia*, 1838.

This essay contains a medical history of emphysema, founded upon the observation of ninety patients, in whom it was detected, in the hospital de la Pitié, at Paris. Forty-two of these died, twenty-three of cholera, and all of affections distinct from the emphysema; the remainder were more or less relieved. Twenty-three of the cases were examined by Dr. James Jackson, Jr. of Boston, to whose perseverance and industry the author pays a just tribute in more than one passage of his treatise. The others were observed by M. Louis himself. So extensive an induction has enabled the author to apply very fully his numerical method and the results are stated with great distinctness and accuracy. We have first a general description of the disease, embracing the prominent symptoms exhibited during life, and the principal appearances presented after death. Then follows a more detailed account, divided into eight sections. The first is headed "anatomical details," and includes a full description of the respective appearances presented by the lungs and heart. The most frequent of these was a dilatation of the vesicles, which in some instances reached the size of a small pea. The tissue of these enlarged vesicles was found to be hypertrophied, in conformity to the general law, by which all the hollow viscera, when their caliber is morbidly enlarged, are found to have their walls thickened. The greatest tendency to dilatation was observed in the free border of the lungs; an argument that emphysema is independent of catarrh, which usually occupies the interior of these organs. Where the vesicles were ruptured, they looked like appendages to the borders of the lungs, and this appearance is alluded to as of very frequent occurrence. Adhesion of the pleura was found in a large proportion of the cases, a circumstance which might at first appear to connect itself, either as a cause or as a co-ordinate effect, with the emphysema; but this idea is opposed, as well by other considerations, as by the fact that the lungs were perfectly free throughout in two out of three cases in which the emphysema was greatest. Whatever the cause of the dilatation, therefore, inflammation of the surrounding textures is not to be regarded as such.

Of the symptoms which accompany emphysema, and which form the subject of the second section, we shall only advert to dyspnœa which is the most interesting, inasmuch as Laennec and others have pretended to discover in emphysema a leading cause of the asthmatic paroxysm. The conclusion arrived at by our author is that pulmonary emphysema may exist without causing dyspnœa, at least for a time, but that such an occurrence is a rare exception to the general rule.

The paroxysms of dyspnœa frequently supervened without any appreciable cause, but most commonly in consequence of an attack of acute pulmonary catarrh. In a single case, they occurred only at night, at which time the patient was obliged to sit up or even go to the window to breathe. As respects the fatal cases, difficulty of breathing was found in all, but paroxysms of dyspnœa were present in three only. Are we by these paroxysms to understand true attacks of asthma? Laennec, as is well known, distinctly maintained that emphysema was the most frequent cause of the latter affection, and that nervous dyspnœa, independent of any organic affection of the lungs, was of rare occurrence. (De l'ausc. §556.) The testimony of J. Jackson, as quoted by M. Louis, is nearly to the same purpose. Out of 120 individuals, of whom he enquired into the state of respiration from infancy, he found twenty-eight who had been subject to, more or less, shortness of breath since that time. Of the latter, one was affected with disease of the heart, two with phthisis, and the remainder with pulmonary emphysema.

Under the head of diagnosis, in section fourth, we are presented with the principal symptoms which mark the affection and which the author considers sufficient to distinguish it from all others. It is described in the following terms:

"A disease without fever, of long duration, which often commences in early youth,* very rarely after fifty years of age, by a slight dyspnœa, which commonly continues without aggravation for a great number of years, when it dates from infancy, afterwards becoming successively more and more marked, and accompanied with paroxysms, during which the patients appear sometimes to be menaced with suffocation. This dyspnœa is frequently preceded by cough, and almost always accompanied, at some period or other of its course, by pulmonary catarrh, which, when aggravated, is one of the most common causes of *paroxysms* of dyspnœa. Connected with these symptoms we find an alteration in the form of the chest, which alteration is generally limited in extent, and consists in a prominence in which both the ribs and intercostal spaces are equally interested, and the most common seat of which is the anterior part of the chest and the supra-clavicular regions. In these elevated portions, the percussion is more sonorous and the respiration more feeble than in the natural condition, and in the other parts of the chest. A sibilant (*sifflant*) or subcrepitant râle is often mixed with the respiratory murmur. In some patients, and at

* This fact has been mentioned by Laennec.

a more or less advanced stage of the disease, we find palpitation, with œdema of the lower extremities. There is no loss of flesh, except when tuberculous disease is also present, or during the course of acute catarrh, which frequently complicates the principal affection, or when the dyspnœa from any cause is great, and continues so for a length of time. Finally, when the patients die, we find, upon opening their bodies, a greater or less dilatation of the pulmonary vesicles."

Emphysema is distinguished from chronic catarrh by the paroxysms of dyspnœa, the prominences of the chest, the *constant* diminution of the respiratory murmur; from dilatation of the bronchia by the weakness of the respiration and the want of resonance in the voice; from tuberculous disease, by the clearness of the sound on percussion and the absence of emaciation, and fever; and from aneurism of the aorta by the absence of the whizzing sound which almost invariably occurs in this affection.

As respects the causes of pulmonary emphysema, which form the subject of the fifth section, it is to be remarked that Laennec regarded catarrh, and the presence of a viscid mucus as among the leading causes of the dilatation of the vesicles. His view of the subject was, that when the vesicles become clogged with their secretions, and are unable to relieve themselves, the inhaled air is imprisoned in them, and that the expiratory muscles being inferior in power to those which maintain the opposite process, are unable to overcome the resistance thus offered. (*De l'ausc.* §262) His explanation is altogether too mechanical, and has beside the disadvantage that if it proves any thing, it proves too much, for in this way the cavity in question must become permanently obstructed, and the return of healthy action to that part of the lungs impossible. But we are informed by M. Louis that, whatever the size of the dilated pulmonary vesicle, even when found as large as a cherry stone, it is quite empty and devoid equally of mucus and false membrane; a fact apparently fatal to M. Laennec's hypothesis. Our author considers the immediate cause of emphysema to be unknown to us, and adds, "do we understand any better the dilatation of the bronchial tubes?" The cause assigned by Laennec is evidently inapplicable here; so that we are compelled to admit both in this case and that of emphysema a force analogous to that which presides over the extension of hollow organs, and in virtue of which the latter enlarge, without our being able to account for it by any obstacle, or other mechanical cause. In regard to remote causes, we are informed, that emphysema is frequently hereditary, and that this influence is much more marked in cases where it dates from early infancy, than in those in which it commences about the age of twenty.

The two remaining sections, on the frequency of emphysema, and on its treatment, require but a brief notice. M. Louis finds the affection of more frequent occurrence than has been supposed. Of fifty patients carried off by cholera, twenty-three were affected with emphysema; yet there is no reason to suppose that any other than an accidental relation subsists between the former and the latter, or that the same number of autopsies of persons equally advanced in life, but dying of different diseases, would have furnished a more favourable result. His views upon the treatment seem to be comprised in the following remarks, which we quote in conclusion.

"If emphysema is simple, mild, that is, accompanied by but little oppression, by slight pulmonary catarrh, with scanty and easy expectoration, without paroxysms of dyspnœa, it is proper—as results from the analysis of the facts previously detailed—to avoid violent emotions, exposure to dust, which I have several times known to bring on paroxysms of oppression, moisture, especially exposure to fogs, which is so often followed by coryza, and afterwards by pulmonary catarrh, or an increase of that which already existed, and consequent paroxysms of dyspnœa. The patient should avoid every thing which quickens the respiration and increases the necessity of breathing, fatigue of body and mind, violent and repeated emotion of all sorts, and too animated conversations."

Dr. Stewardson's translation is expressed in clear and correct language, free from gallicisms, and faithful to the letter as well as the spirit of his original. Having formerly followed M. Louis in Paris, and witnessed many of the cases on which the essay is founded, Dr. S. was particularly well fitted to introduce them to the notice of the American public. E. G. D.

